# Non-Destructive Testing Methods for In Situ Crack Measurements and Morphology Analysis with a Focus on a Novel Approach to the Use of the Acoustic Emission Method

**DOI:** 10.3390/ma16237440

**Published:** 2023-11-29

**Authors:** Grzegorz Świt, Aleksandra Krampikowska, Paweł Tworzewski

**Affiliations:** Department of Strength of Building Materials and Structures, Kielce University of Technology, al. Tysiąclecia Państwa Polskiego 7, 25-314 Kielce, Poland; gswit@tu.kielce.pl (G.Ś.); akramp@tu.kielce.pl (A.K.)

**Keywords:** cracking, non-destructive techniques, construction diagnostics, EA method, digital image correlation, optical sensor, reinforced concrete

## Abstract

This article presents a concise review of modern non-destructive testing (NDT) methods that allow the detection, tracking, and measurement of cracks in reinforced concrete structures. Over the past decades, the range of solutions available on the market has increased. This provides excellent opportunities when choosing and designing systems for diagnosing and continuously monitoring structures. Cracking affects the mechanical properties, durability, and serviceability of a structure or its elements. Therefore, there is a need to develop methods that would allow the determination of the moment of a destructive process’s formation, i.e., a crack’s appearance. At the same time, it is crucial to be able to track the development of cracks for the entire structure, not just selected locations. This work also presents the concept of combining selected NDT methods and creating a system for the continuous monitoring of structural integrity and predicting changes in the durability of existing and future buildings.

## 1. Introduction

The diagnosis and monitoring of the structural integrity of concrete elements is a current and socially relevant issue. Aging infrastructure and growing in-service stresses are the fundamental forces behind the rapid development of a new interdisciplinary field of research strongly related to the durability and safe service life of structural components called structural health monitoring (SHM).

Durability, safety, and serviceability are the three cornerstones of structural reliability, which is understood as the ability of a structure or its components to meet the requirements that have been set in its design. Safety and serviceability are ensured by meeting the load-bearing capacity and serviceability limit states [1,2,3]. Unlike in the past, today, these three elements of reliability have equal significance in the design process.

**Figure 1:**
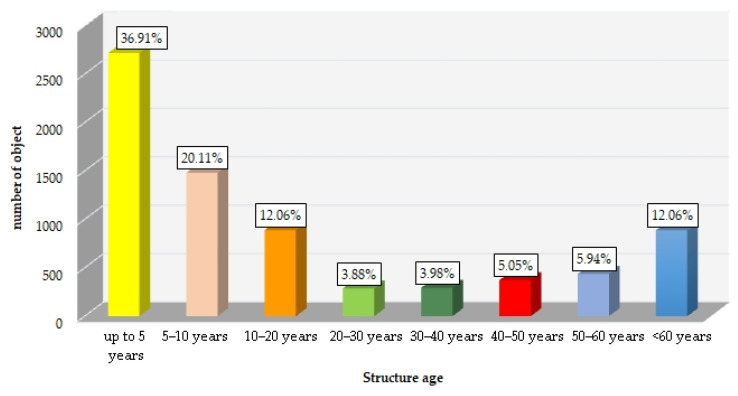
Bridges on national roads managed by the GDDKiA by age [4].

As shown by the GDDKiA data [4] on the age of bridge structures on national roads (Figure 1), more than 60% of bridges were designed according to old standards (which put more emphasis on strength characteristics and less on durability). 

Given that, by 2022, the number of “new” structures increased, as did their age (Figure 1), it is important to determine the environmental impact on the current and future condition of those structures [5].

In order to unify the safety and serviceability provisions of the standards, it has been assumed in Model Code 2010 [6] that they cover both reinforced and prestressed concrete structures. In addition, it was assumed that recommendations are not limited to the useful life of newly designed structures but also include the possibility of estimating the remaining period for existing structures or structures undergoing renovation [6,7].

Underpinning this approach contained in the Model Code 2010 were the following assumptions:

Structural designs are prepared by suitably qualified and experienced designers;

Adequate supervision and quality control are provided in factories and on construction sites;

Structures are erected by qualified and experienced personnel;

Materials and products are used in accordance with the relevant specifications;

Structures will be properly maintained according to standard recommendations;

The minimum requirements for structural workmanship given in EN 13670:2011 [8] are fulfilled.

If we consider the Polish reality regarding the design, execution, use, and maintenance of building structures past and present, we can conclude that these assumptions are too optimistic. It is possible to see in the reality around us a number of structures that have either been poorly designed or in which materials with insufficient parameters for resistance to aggressive environments have been used. The performance of inspections and diagnostics of the condition of a structure by persons without sufficient knowledge, professional experience, or technical capacity cause the inspection documents produced in this way not to be an authoritative or reliable source of information used to determine residual service life.

The research-based recommendations provided in Model Code 2010 [6] indicate three damage mechanisms:

Carbonation-induced corrosion;

Chloride-induced corrosion;

Freezing/thawing.

It can be concluded that the intensity of these three destructive processes is strongly influenced by the formation and development of cracks in a component or a structure [9].

In addition, it has been shown that it is not yet possible to develop new internationally accepted models for other damage mechanisms, e.g., sulfate attacks and the reactivity of alkali aggregates. 

All of these approaches to determining the durability of structures at the design stage of construction and use are applicable in what is known as life cycle management, which requires the production of two documents [6]:

As-built documentation, culminating in issuing a birth certificate document—BCD—containing the results of an initial review of the new design;

A service life file containing a record of all the actions taken during the period of use and the conditions of use, and in the case of major renovation work, it must be supplemented by a re-birth certificate document (RCD).

The term “durability of materials” is used colloquially, although it is not exact. It is particularly important to determine the onset of damage in materials and structural components, as many initiated processes cannot be stopped effectively and economically, leading to the failure of the entire structure.

Therefore, a concept was developed to link the durability limit state not to a conventionally defined degree or extent of failure of a structural element but to the initiation of a failure process triggered via the appearance of cracks that lead inevitably to the occurrence of one of the two traditional limit states [5].

Over the past few decades, a large number of studies have been carried out on the effects of crack width and environmental factors on the corrosion of reinforcement in both non-prestressed and prestressed concrete structures. Some selected results of these studies are presented in Table 1.

The national standards committees and professional organizations and associations on the permissible maximum crack widths that affect the corrosion processes of reinforcement have developed criteria.

The primary conclusion that has emerged from the analysis of the cited studies [10,11] is the need to develop methods that would detect the onset of the formation of destructive processes—the appearance of cracks—as well as track their development and course throughout the entire volume of concrete elements, not only in subjectively selected areas. 

The information on a structure that allows the accurate assessment of its condition includes the location, inventory, and measurement of structural cracks and fractures. The data collection enables the reconstruction of the behavior of a structure and identifies stresses and impacts by evaluating crack morphology, which refers to the width of each crack and its location, organization, and distance from other cracks. In this regard, techniques that enable the computerized surveillance of the technical condition of structures are a step in the building information collection and modeling (BIM) process. 

A variety of methods available on the market allow a solution to be selected to suit the required range of measurements used for building structure diagnosis. These range from simple and inexpensive feeler gauges to sophisticated, continuous structural monitoring systems using acoustic emission (AE) and digital image correlation (DIC).

## 2. Selected Methods for the Measurement of Cracks

### 2.1. Devices Based on Manual Readings without Data Registration

Methods based on manual readings use devices in which the crack width value or its increment is read from a scale by an operator. These simplest methods are cheap and widely used not only in laboratory conditions but also in the field for the diagnostics and monitoring of buildings. They fall into two groups: methods intended for measuring crack widths and methods limited to monitoring changes in the width of existing cracks.

#### 2.1.1. Manual Crack Width Measurement Methods

**Card sizer/feeler gauge, Brinell magnifier, and Brinell microscopes**

The most common method of measuring the crack width in situ involves the use of a length gauge, which must be applied perpendicularly to the crack at the point of measurement [12]. The most commonly used instruments for this purpose include:

A card sizer/feeler gauge with an accuracy of 0.05 to 0.5 mm. It most often finds application in the diagnosis of real-world objects;

A Brinell magnifier—an instrument that provides magnification of up to several times. More commonly used in laboratory research, this magnifier is fitted with millimeter graduations and has an accuracy of 0.1 mm. Some of the more advanced models may have illuminated graduations;

Brinell microscopes—whose accuracy, as well as magnification, is greater than that of magnifiers, e.g., 35–40× magnification and 0.02 mm accuracy, 20× magnification and 0.1 mm accuracy, and 300× magnification and 0.001 mm accuracy. They are typically used in laboratory research.

**Figure 2:**
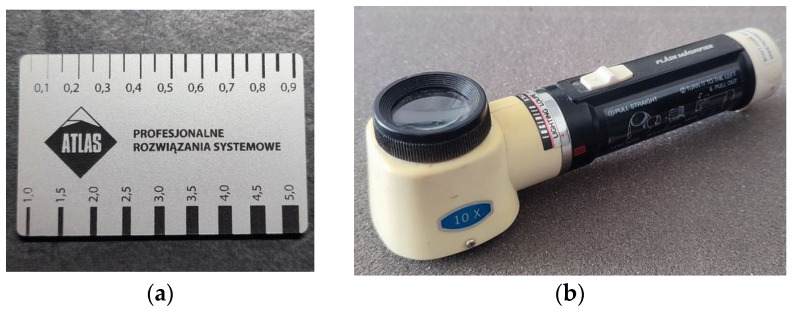

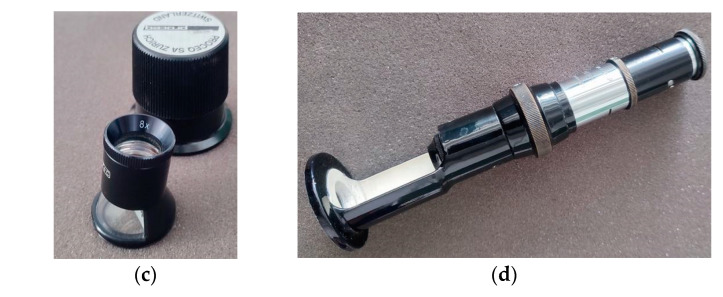
Instruments for measuring crack widths: (**a**) card feeler gauge, (**b**) Brinell magnifier without a backlight, (**c**) Brinell magnifier with a backlight, and (**d**) Brinell microscope.

Magnifying an image improves reading accuracy. If a measurement is to be obtained cyclically, the area to be measured must be properly marked. Moving or rotating the device results in incorrect readings. Examples of instruments [13,14] for classical measurements of crack width are presented in Figure 2.

However, those methods have significant limitations due to the time-consuming nature of the measurements, errors due to fatigue—the influence of the so-called “human factor” on the results—and the unevenness of the surface of the examined element. Correct and legible documentation of the results in the case of a large number of cracks and a large structure is difficult. 

**Figure 3:**
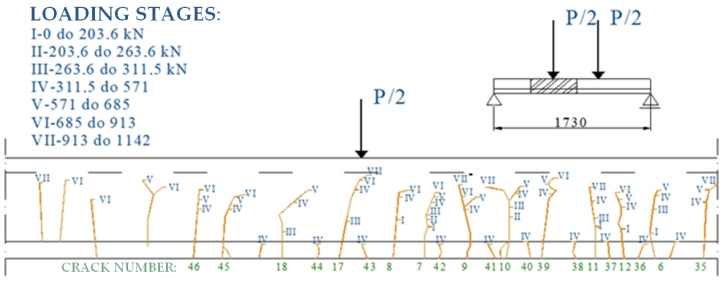
Distribution of cracks on the left side of a KUJAN-NG-type beam until failure.

**Figure 4:**
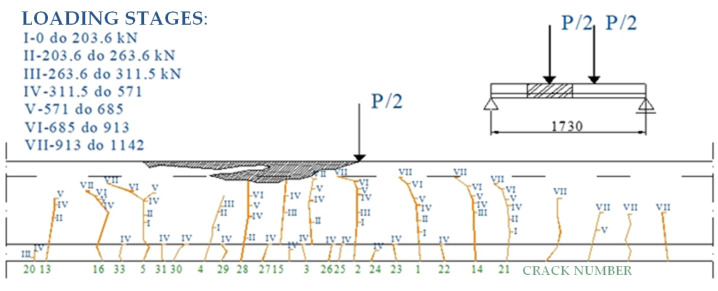
Distribution of cracks on the right side of a KUJAN-NG-type beam until failure.

Crack patterns and propagation are documented as photographs or in the form of a drawing, which takes a lot of time because newly formed cracks and the subsequent growth stages of existing cracks must be marked. Images of a precast, prestressed concrete beam of the “KUJAN-NG” type in the bending test using traditional methods is presented in Figure 3 and Figure 4.

#### 2.1.2. Manual Methods of Monitoring Crack Width Growth

Commercial crack width monitoring and manual reading devices are widely used because of their convenience and low cost. These devices are reusable. They are not intended to measure crack width but, rather, to observe and measure a change. Fully or partially attached to a component, the instrument remains on it.

**Glass or gypsum seals, crack gauge/feeler gauge**

**Figure 5:**
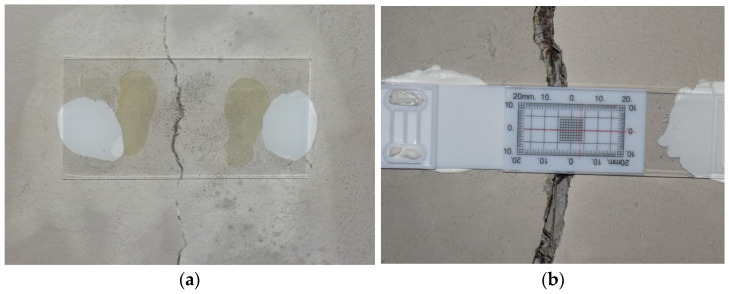
(**a**) Glass seals and (**b**) crack opening indicator.

Traditional methods used to monitor cracking mainly focus on checking whether the damage has expanded and determining the size of the change:

The simplest method—glass or gypsum seals—is a very simple solution consisting of bonding a piece of glass that is 1 mm thick to both sides of the crack or applying gypsum to the crack. A change in the crack width causes the seal to break. No information on the magnitude of the change is provided;

A crack gauge, also known as a feeler gauge (Figure 5), consists of two overlapping plates with a graduation scale. Each plate is fixed on the opposite side of the crack. In addition to noting the change in crack width, the instrument allows its size to be determined to an accuracy of 0.05 mm [13,15]. The instrument usually has three-millimeter divisions. This allows for not only measuring the increase in the crack width but also determining the value of the displacement along the crack and the angle of crack rotation.

**Caliper**

**Figure 6:**
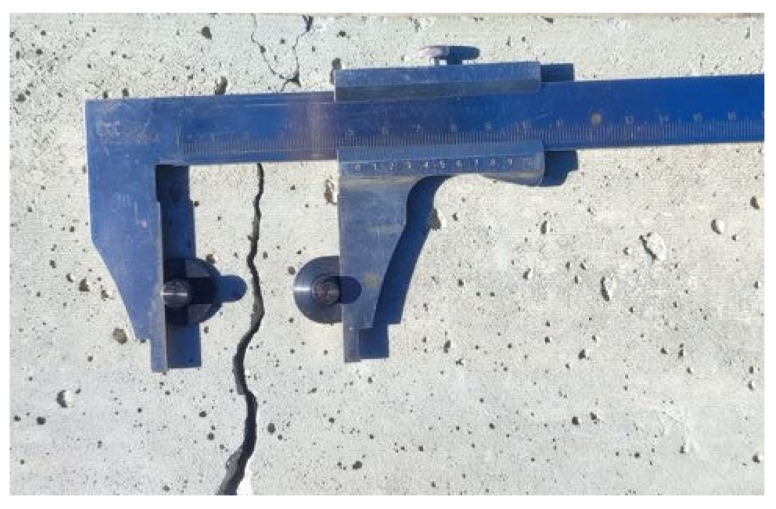
Crack width change measurement using a caliper.

**Figure 7:**
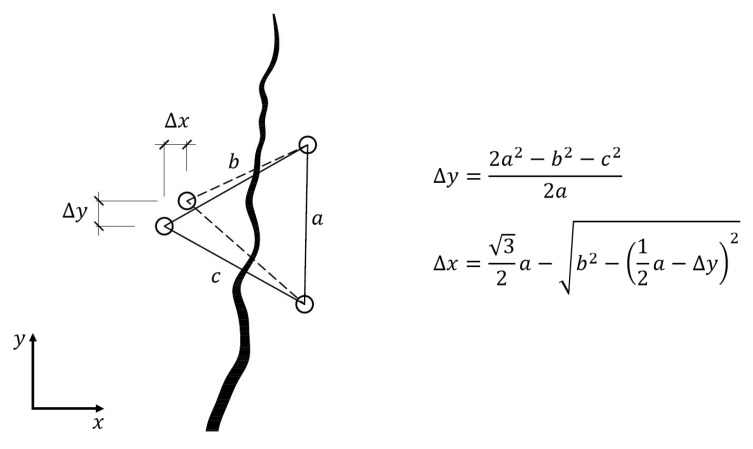
An example crack width change measurement.

A caliper can also be used to monitor crack width changes. To do this, the crack monitoring disks must be glued to the surface or holes must be drilled and pins placed in the holes. The simplest way is to use two disks or pins placed on both sides of the crack so that the line connecting them is perpendicular to the crack. This setting allows the determination of crack width changes (Figure 6). Three or four disks/pins should be used to identify the value of the displacement along the crack and the angle of crack rotation. In the case of a measurement based on three points, two pins/disks should be attached on one side of the crack, parallel to it, and the third one on the opposite side. Their arrangement should create a shape similar to an equilateral triangle. The most commonly used point spacing is 50 mm. In the coordinate system (Figure 7), changes in the Δ*x* value correspond to changes in the crack width, while changes in the Δ*y* value indicate displacement along the crack.

An example of a ready-made solution is the SHM-X system [16], which is based on the use of four points/pins, two of which are placed on one side of the crack and the other two on the other. The included template is used to correctly arrange the pins. To facilitate measurement, a dial or digital caliper can be used. The measurement accuracy depends on the model, but it is usually 0.01 mm.

**Mechanical Strain Gauge**

**Figure 8:**
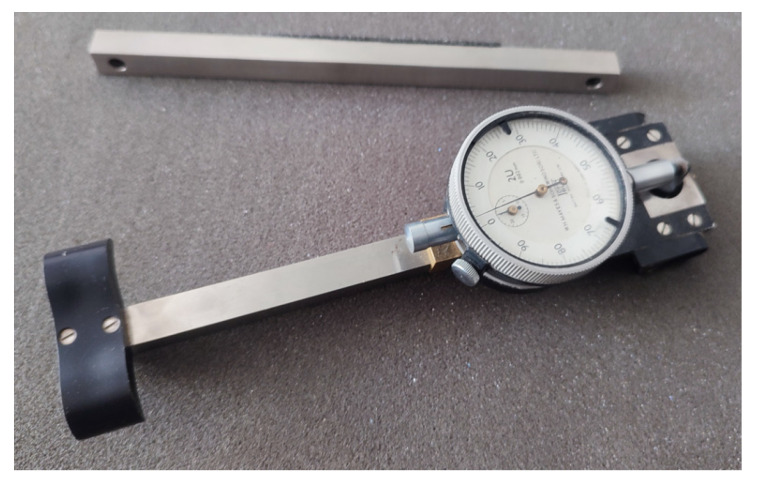
Mechanical strain gauge.

The mechanical strain gauge is used similarly to the caliper (Figure 8). Before measurement, crack monitoring disks should be glued to the surface. To identify the appropriate distance between points, the included template adapted to the measuring base is used. The accuracy of the device is up to 0.001 mm. As with the caliper, digital versions are also available, which make measurements much easier.

### 2.2. Digital Devices with Data Recording

Digital devices and automatic recording help eliminate errors resulting from the “human factor” and shorten the measurement time. As before, there are methods that allow measurements of the width of cracks or those limited to monitoring structure cracks. Typically, these devices are more demanding to use and much more expensive than the devices described above.

#### 2.2.1. Digital Crack Width Measurement Methods

**Optical methods**

These systems use the principles of photogrammetry and various image analysis techniques that allow for the detection of cracks, and generally, they are applicable to many different areas, such as laboratory testing, field inspections, construction quality control, and quality assurance. High-resolution cameras make it possible to detect even small details such as cracks. In the case of most solutions of this type, the person performing the measurement can maintain a safe distance from the surface under testing.

**Automatic crack detection.**

**Figure 9:**
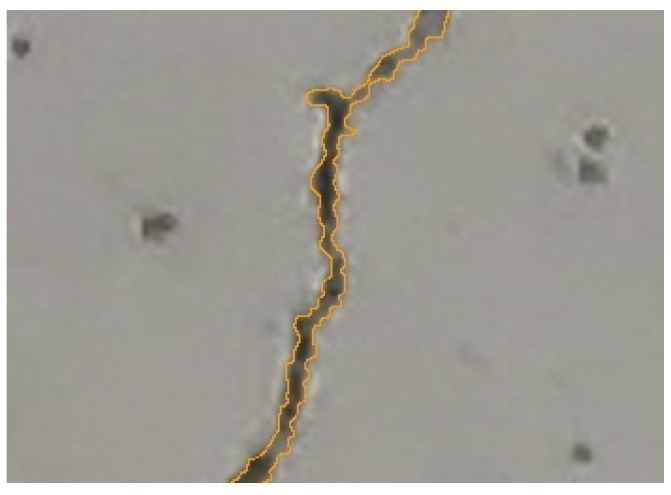
Location of the crack edge based on a digital photo.

In simple terms, this method involves recognizing the color change resulting from the black color of the crack in a digital photo (Figure 9). Examples of various algorithms created for this method can be found in the literature. Such analysis requires good-quality photos, preferably in high resolution. In order for the cracks to be clearly visible in the photo, the surface must be well lit, which may be difficult under outdoor conditions. Moreover, not only the crack may appear black on the surface. Algorithms also often encounter problems in places where concrete crumbles along the length of a crack. Another problem is the measurement of the crack width itself. Calibration is needed for the algorithm to determine the size of the area per pixel. An example solution in the case of two-dimensional measurements is the use of a laser rangefinder to determine the distance from which the photo is taken [17] or detecting the laser beam mark in the image [18]. In the literature, you can find equipment and many algorithms enabling such measurements, from simple ones that allow the analysis of individual cracks [19,20] to solutions that analyze entire elements based on one photo [21,22,23] or many photos integrated into one image [24]. However, ready-made solutions are scarce. 

Numerous crack detection methods are in common use. However, considering the complexity and significance of the matter, the limitations of these techniques often make them inaccurate. Developed in 1986, canny edge detection is one of the widely used methods. It uses a multi-stage algorithm to detect edges in images and Gaussian smoothing to reduce background noise. The deficiencies of the method include oversensitivity to noise, leading to localization errors, and poor adaptability to different conditions. An example of using this method is described in [25].

Otsu’s method (just like the Canny method, categorized as an edge detection method), in the simplest form, automatically separates an image into white and black parts based on the intensity of a pixel against the threshold. It works properly for uniformly illuminated images but performs poorly on underexposed areas in the photo/image. Similar to cracks, the algorithm can assign the same color to this area. An example of using this method is described in [26].

Currently, neural networks (CNNs) or artificial neural networks (ANNs) and machine learning (ML) or deep learning (DL) algorithms are becoming more and more popular in the case of vision-based methods [27]. Unlike the methods mentioned above, in this case, the image is firstly divided into many regions, and in each region, the area considered to be a crack is indicated. Neural networks (CNNs) or artificial neural networks (ANNs) are used to perform crack region classification. However, machine learning (ML) or deep learning (DL) are used for crack detection in selected regions. An example is the use of the combination of ANN and support vector machines (SVM) to identify the separate hyperplane between the crack and backgrounds, described in a previous work [28]. These methods require databases to train and establish network parameters, which is time-consuming. Even though they are much more advanced than those described above, they still do not completely eliminate the problems described earlier.

In the literature, you can find many examples, comparisons, and proposals of vision-based automated crack detection methods [29,30]. Unfortunately, they still struggle with many problems. Further development will create one of the best solutions for detecting, measuring, and monitoring cracks.

An example of a very simple commercially available kit is a digital concrete crack width meter [31]. It includes a camera and a computer with a display. A measurement of the crack width with an accuracy of 0.01 mm is taken automatically from the camera image. The result, in the form of a photo with the graduation and measurement value displayed, can be exported to an SD card. The device has one major limitation, i.e., it only allows single cracks to be monitored or measured.

**Digital image correlation (DIC).**

DIC enables the contact-free recording of changes in the position of any point in space on the surface to be recorded and the creation of deformation or displacement maps of the object under study. An important feature is the ability to track the development and measure changes in the width of cracks. This is possible by juxtaposing consecutive images/measurement steps obtained from two or more cameras. This method, more specifically 2D digital image correlation (2D-DIC), can be considered to have first been used by Sutton et al. in 1983 [32]. The method was later developed into the 3D-DIC method using multiple cameras that capture images at the same time. The history of the evolution of this method is presented in more detail in [33]. The measurement of deformation using the DIC method uses a stochastic contrast pattern, which must first be superimposed on the area under study. The software applies a grid to the recorded image, separating the meshes or facets to which the appropriate grayscale is assigned. The size of the facet is matched to the size of the surveyed area and the resolution of the cameras. The DIC measurement technique is also cost-effective. There is no risk of damage to the components of the apparatus, and the only expense is associated with preparing the tested element for testing by applying the appropriate pattern. Measurements can be carried out with any type of load acting on the structure, either static or dynamic. Optical measuring systems are more and more often complementing or even replacing other devices. Their great advantage is so-called contact-free measurement, a very wide range of measurement possibilities, and the simultaneous acquisition of photographic documentation [34].

The application of this method to the measurement and diagnosis of structures such as bridges or building walls has been described in many publications [35,36,37,38,39,40,41,42]. Typically, the DIC method is used for displacement measurements, as it is not possible to capture a crack when the cameras are positioned at a greater distance from the test object in order to cover a larger area. However, by reducing the distance of the cameras from the object under observation, crack measurement is no longer a problem. An interesting example of the use of digital image correlation is the study of bridge displacements under load from a passing train described in [36].

The Faculty of Civil Engineering and Architecture of the Kielce University of Technology has two digital image correlation systems: the older ARAMIS 5M and the newer ARAMIS SRX. The former uses cameras with a resolution of 2448 × 2050 pixels, allowing for recording at a full resolution of 15 images per second. The system allows the simultaneous use of two sensors/camera sets (2 × 2 cameras), making it possible to record an area up to 4 m wide or two independent areas up to 2000 mm wide. The newer version of the system, ARAMIS SRX, is equipped with cameras with a resolution of 4096 × 3068 pixels, allowing for recording at a full resolution of 75 images per second and a measurement area of 3890 × 3100 mm. Although these systems cover small areas in relation to the size of the entire structure, they make it possible to locate and precisely measure changes in crack width.

**Figure 10:**
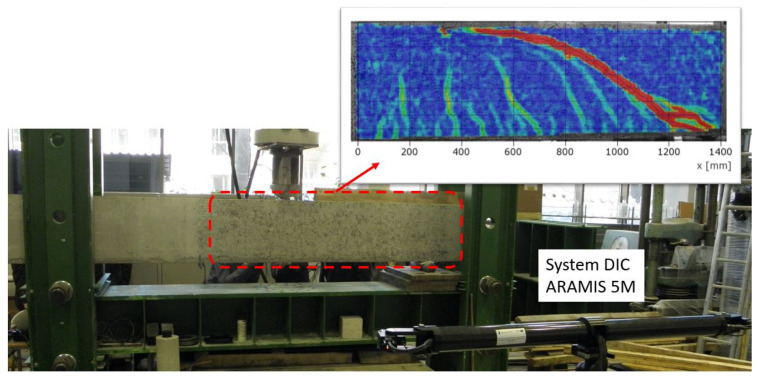
Test stand with the ARAMIS 5M system prepared for measurement and the obtained map of the deformation of the lateral surface of the beam for the marked area.

With the ability to create strain maps for the surface of the component under testing, it is possible to localize cracks and track changes in their width. As an example, the results of measurements carried out on a 0.20 × 0.45 × 6.60 m reinforced concrete beam loaded with a single force monotonically increasing until failure are presented (Figure 10). To force the failure of the element by shear, the point of force application was located near the support. The concrete deformations associated with the formation and development of cracks obtained on the lateral surface of the beams using the ARAMIS 5M system are shown in Figure 10. The crack pattern characteristic of the shear zone is clearly visible, together with the destructive crack.

**Figure 11:**
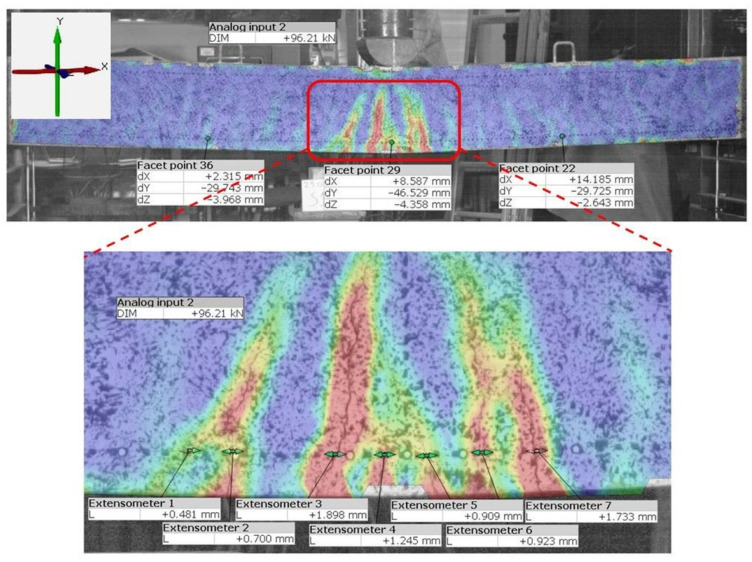
Deformation of the lateral surface of a reinforced concrete beam; results of displacement measurements and crack widths obtained using the ARAMIS SRX system.

Example measurement results obtained using the ARAMIS SRX system are presented for a reinforced concrete beam with a cross-sectional dimension of 0.12 × 0.3 m and an overall length of 3.3 m. A simply supported beam with a span at the support axes of 3 m was loaded with a concentrated force applied at the center of the span. Figure 11 shows a map of the deformation of the lateral surface of the beam and the results of the displacement measurements at three points with individual directions and the width of the crack. The accumulation of deformation shown in Figure 10 and Figure 11 indicates the crack location. Measurements of crack widths were taken using the “Extensometer” function. The measurement accuracy depends on the model, but for the examples described, it was 0.025 mm.

**Fiber optic composite sensors**

**Figure 12:**
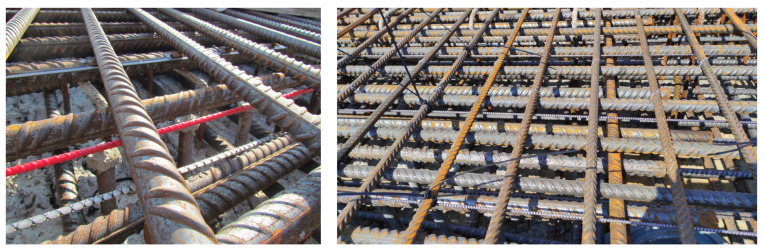
A composite fiber optic sensor and an example of its installation in a reinforced concrete structure (photo by T. Howiacki) [50].

This method enables measurements of deformations, temperatures, displacements, and crack widths, which makes it quite versatile [43,44,45,46,47,48]. Sensors can be installed in newly constructed facilities, for example, by embedding a fiber optic cable in a reinforced concrete structure and concreting it (Figure 12). It is also possible to install it in existing structures, for example by gluing with two-component epoxy glue to its surface. It should be remembered that the measurement is limited to the place where the optical fiber is located; therefore, the correct location is a key element of system design for a given facility. Because a distributed fiber optical sensing (DFOS) device can be placed inside a concrete structure, the method allows for locating internal cracks, which is not possible with optical methods. It is important to select the type of fibers and protective coating in order to obtain the best adhesion when embedded in concrete. During the installation of such a system, you may encounter difficulties related to the possibility of breaking the optical fiber. Deviations in the location relative to the planned location are also a problem. This method is ideal for monitoring large construction facilities. Unlike optical methods, it is almost independent of the influence of external factors.

**Figure 13:**
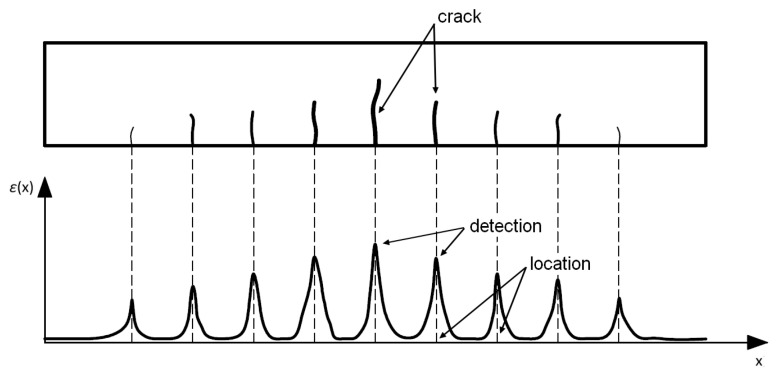
Detection and location of cracks on the DFOS system.

A crack in a reinforced concrete structure causes local discontinuities. As with rebar, this causes strain peaks in the DFOS tool operating within the crack area (Figure 13). With information about strains and the location of the crack along the length of the structure, it is possible to estimate the width of the crack using a method called qualitative analysis. Crack widths can be estimated with sufficient accuracy, i.e., higher than 0.05 mm, using this method.

Because optical fiber slippage may occur, the strain peak is not always large enough to be identified as a crack. Therefore, the algorithms related to correct detection and crack width estimation in this method are constantly being refined [49,50,51].

#### 2.2.2. Digital Methods of Monitoring Crack Width Growth

**Displacement Transducers**

**Figure 14:**
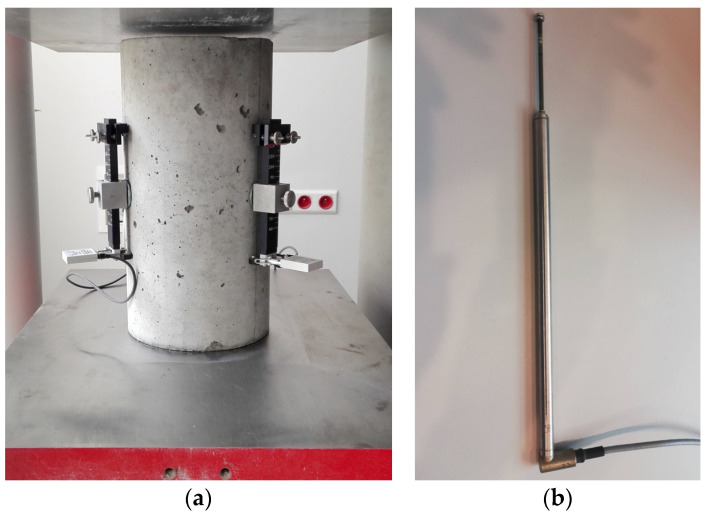
Displacement transducers: (**a**) strain sensors and (**b**) LVDT sensors.

Depending on the required measurement range, strain sensors or LVDT sensors can be used to monitor cracks (Figure 14). In real constructions, LVDT sensors are usually mounted using a spherical plain bearing on pins placed in the drilled holes or disks glued to the surface. As in the case of a caliper, three sensors arranged in the shape of an equilateral triangle can be used for measurements. The sensors must remain on the structure during measurement. They are resistant to dirt, shock, and vibration. The measurement accuracy is usually 0.5% relative to the maximum measuring range of the sensor. Both simple solutions for measuring single cracks and complex multi-channel systems are available on the market [52,53]. Continuous monitoring of the facility is possible using wireless data transmission.

**Magnetic sensor**

The Montec Crack Monitor (MCM) [54] uses patented magnetic technology and allows for the monitoring of crack width and shear. The device does not require an additional power supply because it has a built-in battery lasting at least three years. The data are sent via a mobile phone network and available from any web browser in real time. Like LVDT sensors, this sensor is resistant to weather conditions. The measurement accuracy is 0.025 mm in the X direction (crack width) and 0.05 mm in the Y direction (displacement along the crack).

**Photogrammetric measurements**

**Figure 15:**
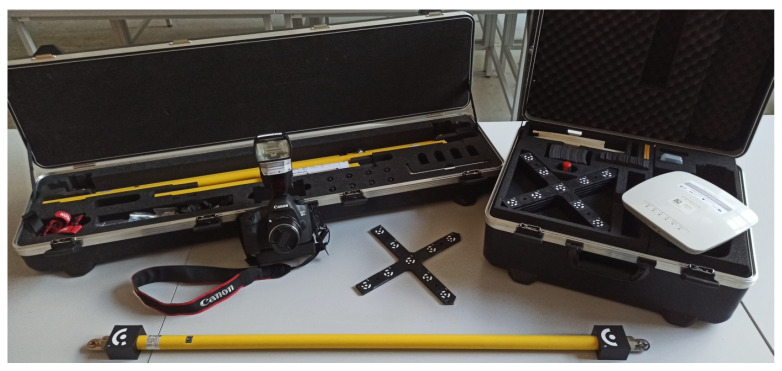
TRITOP system components.

An interesting system that can be used to monitor an existing crack is TRITOP. It is an optical measurement system based on photogrammetry. Its components include a camera, a computer with dedicated software, length standards, and coded and uncoded markers. The main components of the kit are shown in Figure 15. TRITOP allows the verification of the shape and position of the object. The results obtained can be compared with CAD data, i.e., technical drawings. Displacement measurements can be tested when static loads are applied. This is due to the relatively long time it takes to take photographs, particularly for large objects. This method is also suitable for surveillance and monitoring objects, including cracking. It should be emphasized that, for this method, there is no limit to the size of the object being measured, so crack measurements using digital image correlation can be limited to selected areas. At the same time, the rest of the structure can be monitored using the TRITOP system. Repeated measurement is possible at any time by introducing a fixed common coordinate system (determined by coded markers) for both systems so that it remains on the object and does not change its location.

**Figure 16:**
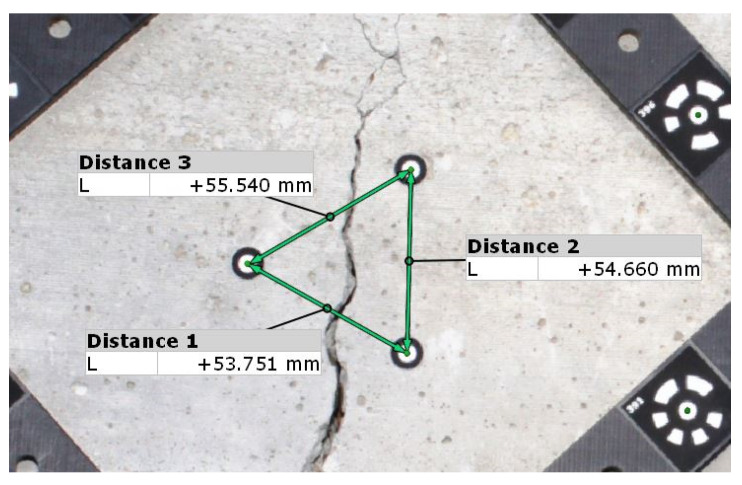
Measuring the length of the sections connecting the points glued around the crack carried out using the TRITOP system.

Preparations for the test consist of laying out the points whose location we want to locate. Non-coded markers are fixed or glued at the locations for which measurements will be taken. If further measurement steps are planned, they are left on the object. In order for the software to be able to locate non-coded markers, coded markers are additionally deployed, which are only distributed at the time of measurement. Some of the coded markers to determine a common coordinate system for subsequent measurement steps should be left on the object. The included length templates allow the software to adjust the appropriate scale. The measurement itself involves taking a “cloud” of images that vary in their angle to and distance from the object. The images are sent wirelessly to the computer’s memory, and the software indicates whether they have been taken correctly. Each tag is given a coordinate position relative to the coordinate system defined by the selected coded tags. Typically, the first stage is the reference. By sticking markers on both sides of the crack, it is possible to monitor and measure changes in the width of the crack on the object. As with other methods, more points can be used in the measurements, which allows for measuring the displacement along the shear crack. An example measurement is shown in Figure 16. During measurement, it is possible to take photos of many places where the points were glued, which allows you to observe a large number of cracks during one stage.

**Figure 17:**
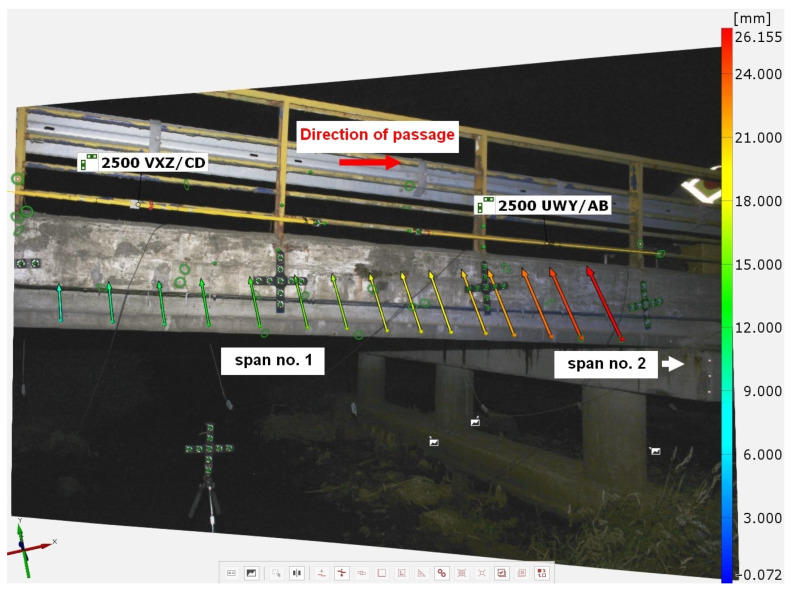
Results of measurements of the displacements of a bridge span after the passage of an oversize transport using the TRITOP system.

Other measurements can also be performed in parallel with crack measurement. As an example of the use of the presented apparatus, measurements of the displacement of one of the two bridge spans after the passage of oversize transport are presented. The position of the glued-on markers on the object was determined for the relieved element. After the transport run, a further step was taken to determine the change in the position of the points and, thus, the deformation suffered by the bridge span. The results in the form of displacement vectors are shown in Figure 17.

Other interesting examples of the use of this system include checking the position of fixing bolts in the foundation of wind turbines to check the fit with the holes in the lowest segment of the tower [55].

Both ARAMIS and TRITOP allow the results to be juxtaposed with CAD data, so the next level is to digitize all the results supplemented by a spatial model. This allows information about the object to be stored and shared digitally. Three-dimensional laser scanning is the ideal solution in this case. It is widely used to inventory urban spaces [56] and building and engineering structures, including structures of historical significance [57], and assess their condition, including deformation [58,59]. The construction models obtained from the scan can be used for quality control, i.e., for a real-time comparison with CAD/BIM files, or to monitor changes to an existing building by comparing successive 3D scan data.

## 3. Selected Methods for Locating Cracks in a Structure

### 3.1. Digital Radiography (X-ray)

**Figure 18:**
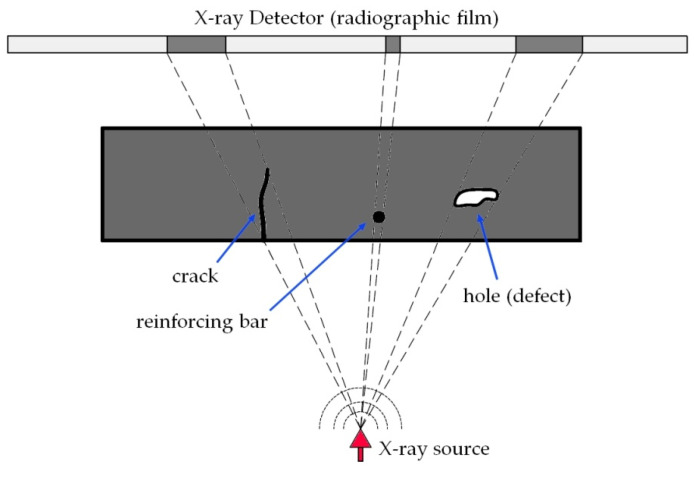
Scheme of using an X-ray system to detect defects in building structures.

The digital X-ray system contains three main parts: an X-ray source, an X-ray detector, and a computer or tablet with controlling software (Figure 18). Systems of this type are mainly used to map the location of conduit, reinforcing steel, and post-tension cables within concrete elements [60]. When it comes to detecting cracks in concrete, this method is mainly applicable to small elements/samples for which it is possible to use X-ray computed tomography (CT) [61,62]. This is due to the high resolution of the image obtained with this method. Under outdoor conditions, the deterioration of construction (which causes changes in the density of concrete) impairs the readability of the X-ray film image. A previous work [63] described the use of X-ray radiography with a contrast medium to locate internal cracks.

### 3.2. Thermography

**Figure 19:**
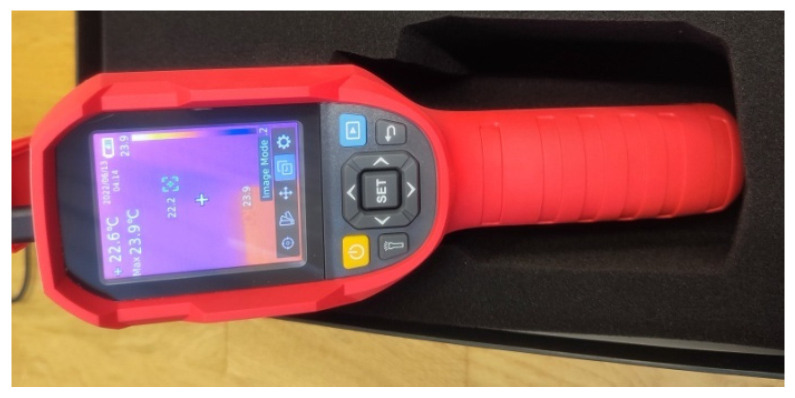
Thermal imaging (infrared) camera.

**Figure 20:**
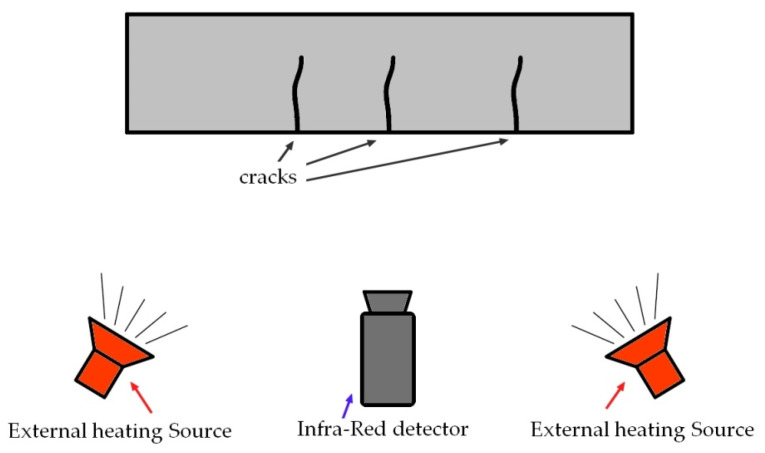
Scheme of using thermography to detect defects in building structures.

Thermography can be used to detect cracks in reinforced concrete structures [12]. Because the method is sensitive to the influence of external conditions such as wind, rain, sunlight, etc., only active thermography can be used for in situ applications. It involves recording with a thermal imaging (infrared) camera (Figure 19) the response of the observed material to the thermal impact generated for this purpose (e.g., using halogen lamps—Figure 20). Another limitation is the small area that can be observed. The method allows the detection of internal cracks, but only at a shallow depth. An example of the use of this method to detect cracks was presented in [64], where it was emphasized that thinner cracks with widths smaller than 0.5 mm can only be observed with additional stimulus (in this case, water). Currently, this method is used to locate defects, including cracks, in elements reinforced using FRP materials [65,66].

### 3.3. Acoustic Methods

#### 3.3.1. Impact Echo Method

Diagnostics of concrete structures using the impact echo method (IE) involves observing the echo of waves reflected at the boundary of the media. The method uses the phenomenon of the propagation of mechanical waves in solid bodies, which are excited by hitting the surface of the tested object with a special hammer. Excitation is performed using a small ball, usually with a diameter of 3 to 20 mm, attached to the end of a flexible arm. The frequency of the generated vibrations is from 10 kHz to 150 kHz and depends on the diameter of the ball used. A graphic recording of the image of an elastic wave propagating in the tested element is made using specialized software. The reflection of elastic waves from the outer edges of the tested element and internal defects in the concrete structure is visible on the frequency spectrum graph in the form of characteristic peaks. This method is used, among others, to determine the thickness of concrete and reinforced concrete slabs accessible on one side or to detect defects in them [12,67,68,69]. The disadvantage of this method is measurement disturbances due to multiple reflections of waves, which makes it difficult to interpret the obtained image in beam elements. The method is also sensitive to the size of the aggregate fraction used in the concrete. According to applicable standards, correction factors should always be introduced to take into account material imperfections [70]. The information we obtain with this method concerns existing damage, but we do not obtain information about the conditions and time of their occurrence. This method is used to detect cracks in reinforced concrete structures only within a small area.

**Figure 21:**
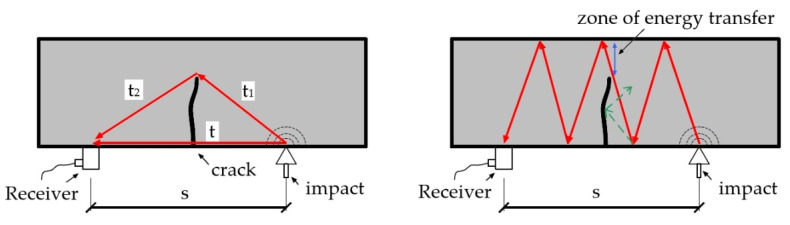
Scheme of using impact echo (IE) method measurements for crack detection.

One way to detect a crack is by using the impact echo method. A crack is a discontinuity in the material that prevents the wave from passing through the shortest path. This causes the arrival time to be longer in damaged areas (Figure 21). Because the area inside the crack is not always filled only with air, correct interpretation can be difficult. In addition, measurements in concrete may be disturbed due to the presence of reinforcing bars. The emitted signal recorded by the receiver can also be used to locate a crack. However, this is only possible in systems where the emitter produces a highly reproducible constant signal. The energy (amplitude) of the recorded signal increases later, and the obtained values are significantly lower in the damaged area because the zone of energy transfer is smaller (Figure 21) [71]. Work is currently underway to automate the measurement and increase the area that can be analyzed using this method and semi-supervised learning [72].

#### 3.3.2. Ultrasonic Flaw Detectors

**Figure 22:**
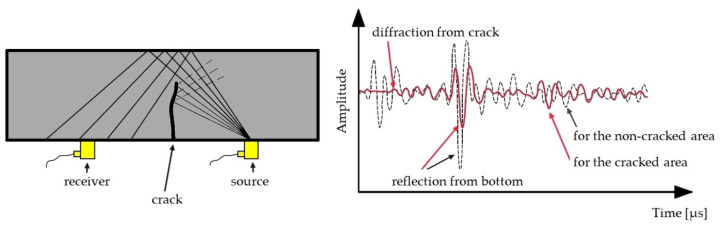
Detection of a concrete surface crack using the ultrasonic method.

**Figure 23:**
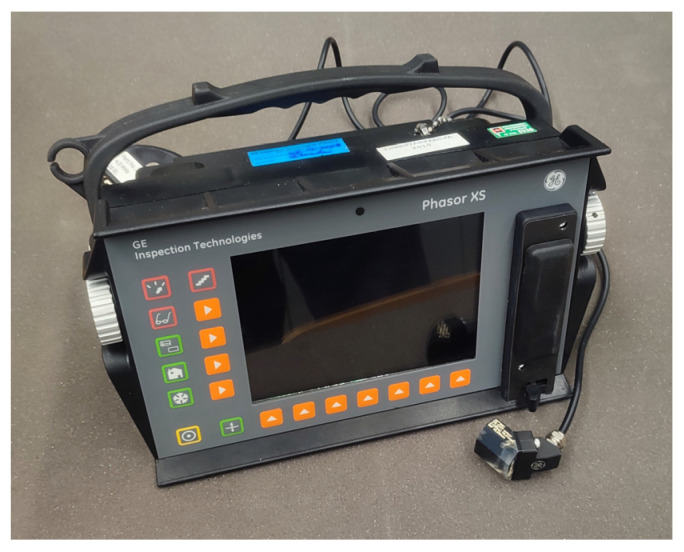
Ultrasonic flaw detector.

Ultrasonic flaw detectors make it possible to determine the wave velocity and its changes in a cross section of an element [12,73,74]. The device produces periodically repeating ultrasonic pulses of longitudinal waves and receives them after passing a known path. The recorded signal is presented in the form of an amplitude–time graph (Figure 22), where the size of the discontinuity is determined based on the amplitude value of the received echo, and the depth of the defect location is determined from the location of the echo on the flaw detector’s time axis. This type of test can be used to locate reinforcement in the cross section, and the gradient of velocity changes allows the estimation of the reinforcement diameter. Based on the types of waves used, the method enables the detection of discontinuities (cracks) in the structure of concrete elements and their location, dimensions, orientation, and nature. An example device is shown in Figure 23.

The definite advantages of ultrasonic flaw detection are their speed of diagnosis, direct access to results, universality, high efficiency, compact equipment, and ability to precisely determine the location of defects.

The disadvantages of the method include the need to have appropriate qualifications to calibrate the device and interpret the results, difficulties in examining elements of small dimensions, and the poor quality of results for coarse-grained and non-homogeneous surfaces. Tests often require the use of a so-called coupling layer, which facilitates the wave’s passage between media by eliminating the air between them. There are limits to the size of the assessed defects, depending on the selected measuring head. Depending on the device model, limitations are related to the maximum observation range, sensitivity (the size of defects as a function of the distance from the head), the dead zone, the resolution, and the observation range.

#### 3.3.3. The Acoustic Emission Method (EA or AT)

This method is a tool that enables early detection of cracks. It is based on the phenomenon in which high-frequency ultrasonic waves are generated as a result of the sudden release of energy inside the material, for example during the initiation and development of cracks.

As mentioned earlier, research and implementation work involves looking for a structural monitoring system that is capable of detecting the start of the deterioration process, as well as tracking its development and progress throughout the entire volume of the structure and not just in subjectively selected areas. Such a method is the acoustic emission method based on the analysis of active destructive processes [75,76].

The results of a study on the application of the identification of active destructive processes (IADP-BIS) method [5] to localize and identify destructive processes, track development, and determine approximate crack widths based on the acoustic emission method are presented below.

**Figure 24:**
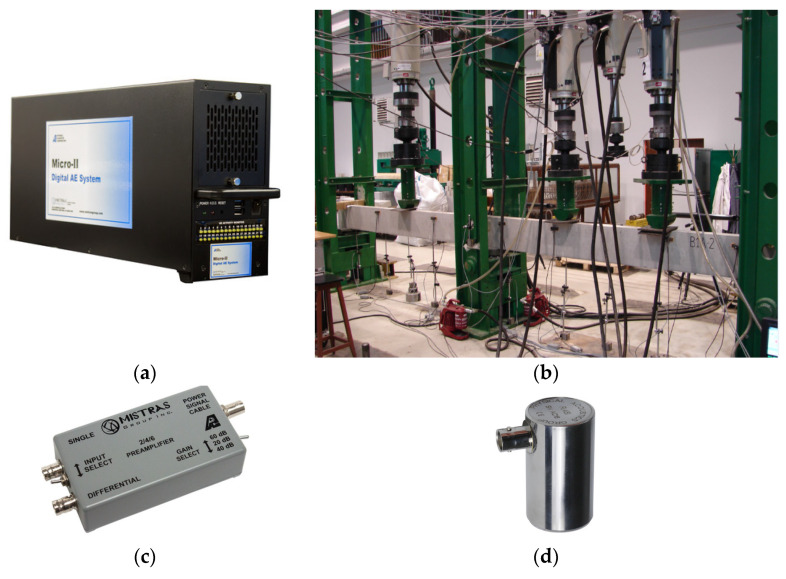
Measurement system for the location and identification of cracks based on the acoustic emission method: (**a**) acoustic emission processor, (**b**) measurement system, (**c**) preamplifier, and (**d**) EA sensor.

Testing was carried out using the acoustic emission (EA) measurement set presented in Figure 24, consisting of the following elements: a 24-channel acoustic emission system with a PC for recording and processing EA signals and their parameters; preamplifiers enabling the transmission of signals at distances of up to 150 m from the measuring site; piezoelectric resonance acoustic emission sensors with frequencies of 55 kHz or with flat characteristics in the range of 30–80 kHz; brackets for mounting the sensors equipped with flexible inserts, enabling the appropriate clamping of the sensors; a device for the recording, processing, and visualization of measurement results (recorded measurement data); a set for numerical analysis of EA signals, enabling the classification and localization of EA sources, e.g., NOESIS 12.0; and NOESIS 12.0 and a database of reference signals.

In addition, verification of the measured crack widths on reinforced concrete beams was performed using an ARAMIS-type optical strain measurement system, as discussed in [75,77,78].

**Figure 25:**
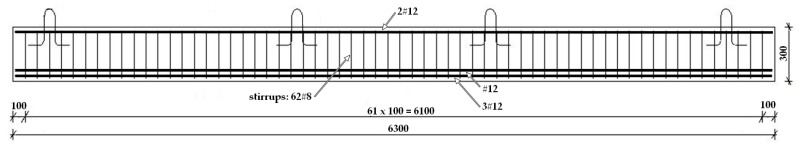
Reinforcement of a reinforced concrete beam [5].

The feasibility of correlating EA signals with crack width, taking into account the reference signal base contained in the IADP-BIS method, is presented below using a single beam as an example (38 beams with different reinforcement layouts, reinforcement levels, and concrete classes were used in the validation study; fifteen bridges and four buildings with a frame structure were examined). In the course of laboratory and “in-situ” tests to date, it was noted that the appearance of individual classes only indirectly depends on the strain of the concrete elements tested and is more related to execution errors, technological errors, and hidden defects; as a result, owing to the EA method, we are informed earlier about the location and type of the destructive process. Therefore, the load levels, as well as the values of the bending moments, are not given in the examples presented below. The test was carried out on a two-span reinforced concrete beam made of class C40/50 concrete over basalt aggregate and class A reinforcing steel. The overall length was 630 cm, the width was 12 cm, and the height was 30 cm. Figure 25 shows the beam with its reinforcement.

**Figure 26:**
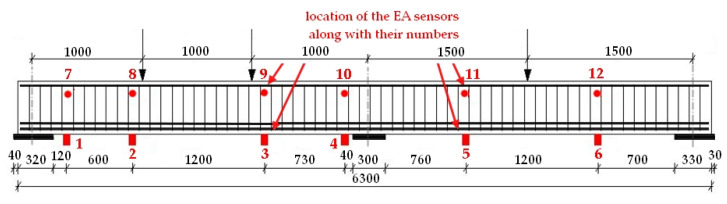
Schematic diagram of a reinforced concrete beam loading rig with sensor arrangement [5].

During testing on an INSTRON—1000-kN-type testing machine, the beam was loaded with three concentrated forces. The test rig for the reinforced concrete beam with the location of the EA sensors is shown in Figure 26.

**Figure 27:**
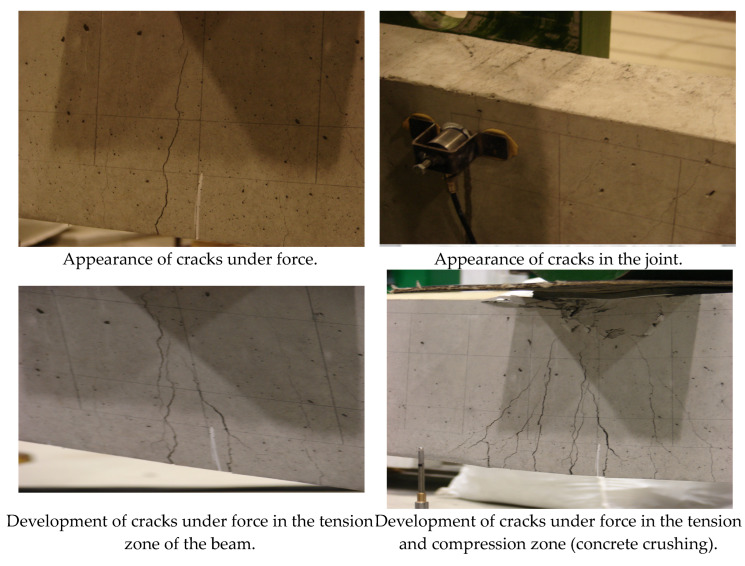

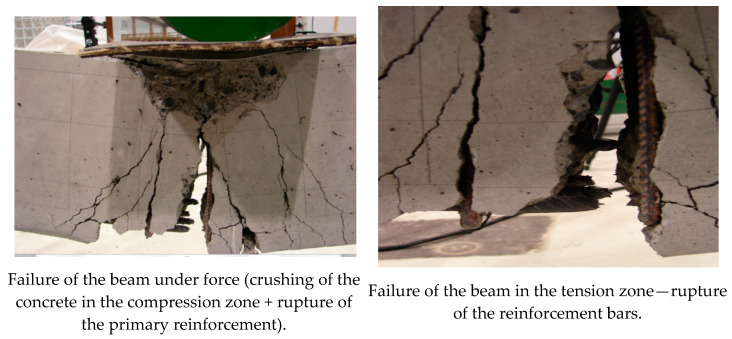
Development of cracking in the tension and compression zone, together with the rupture of reinforcing bars [5].

Figure 27 presents photographic documentation of the resulting cracks and damage location.

It is worth noting that here we have a classic model of the failure of a continuous beam, with cracking appearing in the tension zone under the loading forces, as well as at the intermediate support. The test was conducted until the full failure of the beam occurred in the span under a single force by crushing the concrete in the compression zone and breaking the reinforcing bars in the tension zone.

The acoustic emission signals generated via the processes accompanying the loading of the beam were recorded during the study, which were then subjected to SPR (supervisor pattern recognition) analysis using the IADP-BIS pattern signal database [5].

The affiliation of the signals shown on the graph to the respective classes is indicated using the corresponding color and shape of the point according to Table 2.

**Figure 28:**
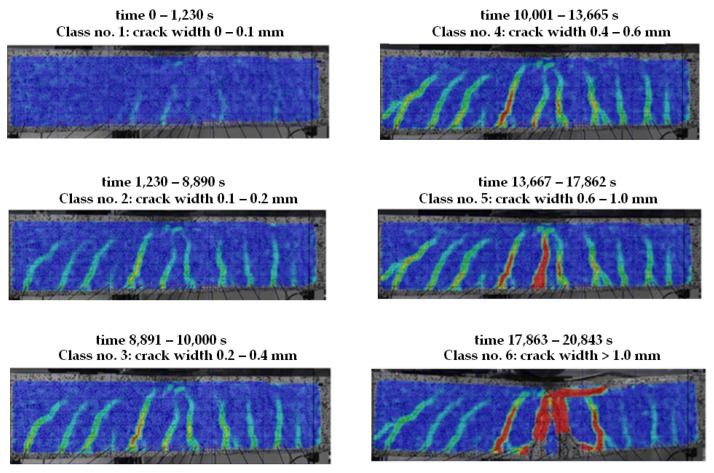
Locating and correlating crack opening widths measured with an ARAMIS-type optical measurement system with the acoustic emission signal classes defined in the reference signal database of the IADP-BIS method [5].

**Figure 29:**
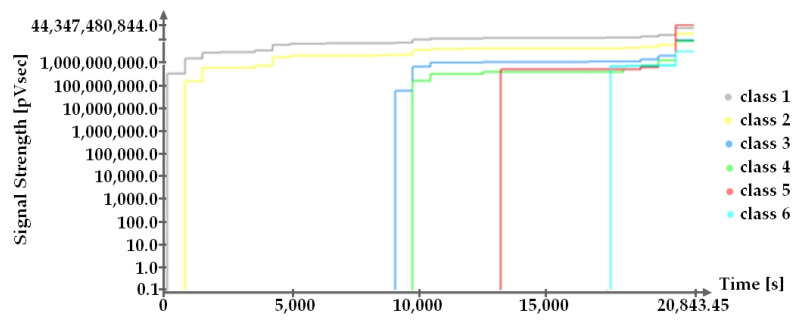
Summary diagram of EA signal strength on a logarithmic scale as a function of time [5].

Class affiliation is characterized by the damage mechanism and hazard code. The dynamics of these processes are correlated with the crack width measured at the respective load levels using an ARAMIS-type optical measurement system and presented in Figure 28. On the other hand, the activity of the destructive process changed, and their identification with the previously presented class numbers (Table 2) is presented in Figure 29, describing the changes in EA signals in the zone between sensors 5, 6, 11, and 12 distributed according to Figure 26 [5].

The maximum recorded crack width was 4.1 mm for crack number 11.

At the same time as measuring the width of the cracks, the acoustic emission signals that accompanied the damage formation were also recorded. The appearance of each destructive class is shown in the summation diagram of EA signal strength on a logarithmic scale as a function of time in Figure 26.

When comparing Figure 28 and Figure 29, it is important to note that the appearance of the individual destructive classes corresponds to the point at which the width of the measured cracks reached the assumed values.

On the basis of our own research [5] on prestressed and reinforced concrete beams and real structures of bridges and timber-framed buildings, as well as the analysis of results in papers [75,76,77,78,79,80,81,82] using reference signal databases developed for methods based on the acoustic emission method, the criteria for the estimation of crack widths were developed using the recording of acoustic emission signals generated during the operation of the test object. The criterion indicates the code and description of the hazards, depending on the class of reference signals describing the destructive processes in the concrete element under investigation and the crack widths assigned to these classes. These criteria are included in Table 3 [5].

## 4. Conclusions

There are many solutions available on the market that can be used to measure and monitor cracks in reinforced concrete structures. All methods have their advantages, disadvantages, and limitations. This, of course, affects the selection of the solution to the requirements and conditions planned for obtaining the measurements. For the diagnosis of small objects with little damage, without the need to monitor cracks, the best choice is simple, cheap solutions, i.e., devices based on manual reading without data registration, such as a magnifying glass or a card. If the number of cracks is significant, the use of digital equipment is a great help, and optical methods are the best solution here. The most promising solution is the automatic crack detection method. The automatic detection and measurement of cracks, combined with the possibility of recording larger surfaces using ordinary digital cameras, is a perfect combination. This method also has great potential for monitoring objects. Digital image correlation is a very good solution, but its biggest disadvantage is the need to prepare the surface and perform a zero measurement as a reference point, which is not always possible under in situ conditions. If we want to monitor the increase in crack width and, at the same time, measure structure displacements, photogrammetric methods (such as TRITOP) are a very good solution.

In the case of the continuous monitoring of large objects such as bridges, wireless communication and a wide range of information obtained are of great importance [83]. The combination of the EA and DFOS methods eliminates the limitations of individual systems. The EA method detects and locates a crack and provides information on what may be the cause of the defect, while in selected places, DFOS allows for measurements of the crack width and element deflection. Each destructive process is a source of acoustic emission, which is characterized by the descriptors of the recorded signal. By linking destructive processes to crack widths, it is possible to assess their impact on safety by monitoring and making observations of the structural health on an ongoing basis and predicting changes in durability, especially that of structures built and designed between 1945 and 2005, for which we do not have full knowledge of the loading history.

In the case of smaller reinforced concrete objects, optical measurement systems gain an advantage over DFOS. They are cheaper, are ideal for measurements in smaller areas, and do not have to be permanently installed on the structure. In such a case, the EA system is responsible for continuous monitoring, while optical systems are used to confirm and measure cracks in places where destructive processes are located. It should be emphasized that it is worthwhile to control the deflection of the tested elements in addition to the crack width, and in continuous beams, the angle of rotation over the support is also important, in addition to the deflection. Detailed ranges are given in Eurocode 2, and here, deformation-measurement-based systems (DIC and photogrametry) are helpful.

The ARAMIS and TRITOP systems allow the results to be juxtaposed with CAD data, so the next level is to digitize all results supplemented by a spatial model. This allows information about the object to be stored and shared digitally. Three-dimensional laser scanning is the ideal solution in this case. It is widely used to inventory urban spaces and building and engineering structures, including structures of historical significance, and assess their condition, including deformation. The combination of an acoustic emission system based on signal analysis using the IADP-BIS database [5] and the ARAMIS and TRITOP systems make it possible to identify, based on the scanning of the structure model, the location and measurement of cracks, to supervise changes in the technical condition of the structure in real time with CAD/BIM files, or to monitor changes in the existing structure through a comparative analysis of subsequent 3D scanning data, as assumed in the BIM.

A comparison with a description of the advantages and disadvantages of individual methods is presented in Table 4 and Table 5.

## Figures and Tables

**Table 1:** Evaluation of the influence of crack width on the corrosion of reinforcement according to selected researchers [5].

No.	Researcher	Crack Width	Conclusions
Short- and long-term tests of non-prestressed concrete structures			
1.	Houston, Atimtay, and Ferguson [8]	0.13 mm	Critical crack value with 25 mm lagging
2.	Okada and Miyagawa [9]	0.10–0.20 mm	Critical crack value
3.	Swamy, R.N. [10]	0.10–0.15 mm	Critical crack value
4.	Vennesland and Gjorv [11]	0.40–0.50 mm	Critical crack value
5.	O’Neil, E.F. [12]	0.40 mm	Critical crack value
Short-term tests of prestressed concrete structures			
6.	Poston, R.W. [13]	0.05 mm 0.38 mm	No corrosion of the reinforcement Same corrosion course in both types of reinforcement
7.	Moore, Klodt, and Hansen [14]	0.10 mm	Minimum width of cracks where corrosion was observed
8.	Perenchio, Fraczek, and Pfiefer [15]	0.25 mm	Lagging thickness of 25 mm is not sufficient protection against the corrosion of beams with and without cracks

**Table 2:** Characteristic signal classes assigned symbols, colors, and codes [5].

Class No.	No. 1	No. 2	No. 3	No. 4	No. 5	No. 6
Color/symbol	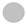	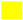	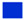	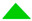	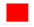	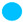
Hazard code	5	4	3	2	1	0

**Table 3:** Criteria for evaluating hazard codes, EA signal class no., and crack widths [5].

Code	Description	Class No.	Crack Width [mm]
0	Element not fulfilling its utility function or damaged	No. 6	>1.0
1	Serious defect/damage and/or element is close to failure/destruction	No. 5	0.6–1.0
2	Moderate defect/damage that may affect load bearing capacity	No. 4	0.4–0.6
3	First signs of structural deterioration; small defects/damage appear which do not affect the load-bearing capacity of the element	No. 3	0.2–0.4
4	New element or element with a defect that does not impair load-bearing capacity	No. 2	0.1–0.2
5	New element without defects—SLS-compliant	No. 1	0–0.1

**Table 4:** Comparison of methods for the measurement of cracks.

Method	Measurement Accuracy	Advantages	Disadvantages
Manual crack width measurement methods			
Card sizer/feeler gauge	0.05 to 0.5 mm	Easy to use;	Low accuracy;
		Cheap.	in the case of many cracks, measurements are time-consuming.
Brinell magnifier and Brinell microscopes	Depending on the magnification of the instrument, 0.1 mm to 0.001 mm	Easy to use;	In the case of many cracks, measurements are time-consuming and tiring for the researcher (may result in erroneous readings);
		Cheap;	In the case of repeated measurements, the instrument should be placed perfectly in the same place;
		High accuracy for Brinell microscopes with higher magnification.	Uneven edges may affect the crack width reading.
Manual methods of monitoring crack width growth			
Glass or gypsum seals	-	Easy to use;	It only informs about changes in crack width; it does not measure this value;
		Cheap.	The shrinkage of glue or gypsum during setting may lead to the cracking of the seal.
Crack gauge/feeler gauge	0.05 mm	Easy to use;	Point measurements in predefined locations.
		Cheap;	
		Allows measurements of changes in crack width, displacement along the crack, and the angle of crack rotation.	
Caliper	0.01 mm	Easy to use;	Point measurements in predefined locations.
		Cheap;	
Mechanical strain gauge	0.001 mm	Depending on the number of disks or pins used, it allows measurement of not only changes in crack width but also displacement along the crack and the angle of crack rotation;	
		Does not require leaving equipment in the monitored facility.	
Digital crack width measurement methods			
Automatic crack detection methods	Depends on the resolution of the cameras and the distance from the recorded object	Measurements possible in any location on the surface observed via a camera;	Requires good-quality photos, preferably in high resolution;
		Can be used both under laboratory conditions and in situ;	Cracks should be clearly visible in the photo;
		Automatic detection;	The surface must be well lit;
		The possibility of recording large surfaces;	Dark areas on the surface can be recognized as cracks;
		Uses ordinary digital cameras;	The method is still under development.
		Crack patterns and propagation are documented as photographs.	
Digital image correlation (DIC)		Measurements possible in any location on the surface observed via cameras;	Appropriate surface preparation is required;
		Can be used both under laboratory conditions and in situ;	Measurements can be carried out with any type of load acting on the structure, either static or dynamic;
		In addition to crack width measurements, it allows measurements of any displacements anywhere in the analyzed area;	In order to measure the crack width, it is necessary to take photos/stage 0 before the damage occurs;
		Crack patterns and propagation are documented.	Expensive equipment.
Fiber optic composite sensors	Higher than 0.05 mm	Enables the detection and localization of cracks occurring along the length of the installed optical fiber;	Expensive equipment;
		Enables measurements of deformations, temperatures, displacements, and crack widths;	Because optical fiber slippage may occur, the strain peak is not always large enough to be identified as a crack;
		An ideal method for monitoring large objects, such as bridges;	The type of fibers and protective coating must be selected correctly in order to obtain the best adhesion when embedded in concrete;
		Can be used both under laboratory conditions and in situ;	The measurement is limited to the place where the optical fiber is located;
		Allows for the detection of cracks inside the structure.	The need to leave the equipment at the monitored facility and have a constant power supply.
Digital methods of monitoring crack width growth			
Displacement transducers	Usually, 0.5% relative to the maximum measuring range of the sensor	High accuracy;	Point measurements in predefined locations;
		Enables measurements of displacements;	The need to leave the equipment at the monitored facility and have a constant power supply.
		Extended configurations allow measurements of changes in crack width and displacement along the crack and the angle of crack rotation;	
		Automatic measurements and data transmission via the internet;	
		The possibility of using multiple sensors.	
Magnetic sensor	0.025 mm in the X direction (crack width) and 0.05 mm in the Y direction (displacement along the crack)	Allows measurements of changes in crack width and displacement along the crack;	Point measurements in predefined locations;
		Battery power supply;	The need to leave the equipment at the monitored facility.
		Automatic measurements and data transmission via the internet;	
		The possibility of using multiple sensors.	
Photogrammetric measurements	Depends on the resolution of the cameras and the distance from the recorded object	Allow measurements of changes in crack width and displacement along the crack and the angle of crack rotation;	Requires attaching/sticking markers to the object;
		The possibility of taking measurements at many points at one time;	Point measurements in predefined locations.
		Do not require leaving equipment on the monitored facility;	
		No remote measurements are possible.	

**Table 5:** Comparison of non-destructive methods for detecting defects in RC structures.

Method	Advantages	Disadvantages
Impact echo and ultrasonic method (UT, UT-PA, and TOFD)	The detection of internal defects in concrete;	Measurements should not be taken directly in cracked, cancerous, or corroded places, in the immediate vicinity of reinforcing bars, or in areas of the highest stress concentration;
	Determines the wave velocity and its changes in the cross section of the element;	Small detection area;
	Allows the determination of crack depth;	The method is sensitive to, among other things, differences in the moisture content of the concrete surface and the presence of reinforcing bars.
Acoustic emission method (AT)	Can measure the entire structure;	AE signal attenuation;
	Temporary and continuous monitoring;	Sensitivity to equipment setting errors;
	Location of cracks;	Sensitivity to the incorrect selection of sensor frequencies.
	Identification of the destructive process;	
	Crack development;	
	Identification of discontinuities in the material structure;	
	Monitoring the reinforcement corrosion process;	
	Estimation of crack widths;	
	Assessment of the intensity of the development of destructive processes;	
	Detection of damage in inaccessible places.	
Digital radiography (X-ray)	Systems of this type are mainly used to map the location of conduit, reinforcing steel, and post-tension cables within concrete elements;	The deterioration of construction (causes changes in the density of concrete) impairs the readability of the X-ray film image.
	Can be used to detect cracks in concrete (mainly applicable to small elements);	
	X-ray radiography can be used with a contrast medium to locate internal cracks.	
Thermography	Can be used to detect cracks in reinforced concrete structures.	This method is sensitive to the influence of external conditions such as wind, rain, sunlight, etc.;
		Only a small area can be observed;
		The method allows the detection of internal cracks, but only at a shallow depth;
		Thinner cracks with widths smaller than 0.5 mm can only be observed with an additional stimulus.
